# Advancing risk factor identification for pediatric lobar pneumonia: the promise of machine learning technologies

**DOI:** 10.3389/fped.2025.1490500

**Published:** 2025-03-07

**Authors:** Li Shen, Jiaqiang Wu, Min Lu, Yiguo Jiang, Xiaolan Zhang, Qiuyan Xu, Shuangqin Ran

**Affiliations:** ^1^ Department of Pharmacy, Suzhou Hospital, Affiliated Hospital of Medical School, Nanjing University, Suzhou, Jiangsu, China; ^2^ School of Life Sciences and Biopharmaceutical Science, Shenyang Pharmaceutical University, Shenyang, China; ^3^ Department of Pediatric, Suzhou Hospital, Affiliated Hospital of Medical School, Nanjing University, Suzhou, Jiangsu, China

**Keywords:** lobar pneumonia, machine learning, risk factor, pediatric, XGBoost

## Abstract

**Background:**

Community-acquired pneumonia (CAP) is a prevalent pediatric condition, and lobar pneumonia (LP) is considered a severe subtype. Early identification of LP is crucial for appropriate management. This study aimed to develop and compare machine learning models to predict LP in children with CAP.

**Methods:**

A total of 25 clinical and laboratory variables were collected. Missing data (<2%) were imputed, and the dataset was split into training (60%) and validation (40%) sets. Univariable logistic regression and Boruta feature selection were used to identify significant predictors. Four machine learning algorithms-Logistic Regression (LR), Support Vector Machine (SVM), Extreme Gradient Boosting (XGBoost), and Decision Tree (DT)-were compared using area under the curve (AUC), balanced accuracy, sensitivity, specificity, and F1 score. SHAP analysis was performed to interpret the best-performing model.

**Results:**

A total of 278 patients with CAP were included in this study, of whom 65 were diagnosed with LP. The XGBoost model demonstrated the best performance with an AUC of 0.880 (95% CI: 0.807–0.934) in the training set and 0.746 (95% CI: 0.664–0.843) in the validation set. SHAP analysis identified age, CRP, CD64 index, lymphocyte percentage, and ALB as the top five predictive factors.

**Conclusion:**

The XGBoost model showed superior performance in predicting LP in children with CAP. The model enabled early diagnosis and risk assessment of LP, thereby facilitating appropriate clinical decision-making.

## 1 Introduction

Community-acquired pneumonia (CAP) is a common respiratory infectious disease in children and the leading cause of pediatric hospitalization, posing a significant threat to the health of children under 5 years of age (1, 2). It has been reported that there are approximately 120 million new cases of community-acquired pneumonia in children each year globally, resulting in nearly 1 million deaths among children under 5 years old (3). Among all CAP cases, lobar pneumonia (LP) represents a severe subtype associated with higher rates of complications and mortality (2). However, the clinical symptoms and signs of LP are non-specific (4), making definitive diagnosis primarily dependent on chest x-ray or CT imaging. Early-stage imaging studies often struggle to provide a conclusive diagnosis of LP. Moreover, both domestic and international guidelines advise against the routine use of chest imaging as a standard diagnostic tool for pediatric CAP (5, 6). Consequently, given the potential severe outcomes, identifying risk factors for LP in children is of paramount importance.

In recent years, machine learning methods have demonstrated significant potential in predicting CAP outcomes and identifying high-risk patients. Xu et al. (7) successfully predicted adverse outcomes in CAP patients using various machine learning algorithms, with the random forest model performing best, achieving an accuracy of 84.9%. In another study (8) developed a deep learning-based model capable of accurately predicting survival in CAP patients using commonly available clinically relevant feature variables, achieving an AUC of 0.917. A machine learning-based causal probabilistic network model demonstrated superior performance in predicting 30-day mortality for adult patients with community-acquired pneumonia compared to existing clinical scoring systems (such as CURB-65) (9). Among various machine learning algorithms, XGBoost has shown exceptional performance in medical applications. Recent studies have demonstrated XGboost algorithms superior predictive capabilities compared to other algorithms. For instance, in predicting COVID-19 severity, XGBoost achieved an AUC of 0.93, outperforming Random Forest (AUC = 0.89) (10). Additionally, Zhou S. et al. developed an XGBoost-based machine learning model that not only achieved excellent performance in predicting the 28-day mortality risk for SIC patients (AUC up to 0.923) but also provided personalized prediction explanations through SHAP analysis (11).

**Figure 1:**
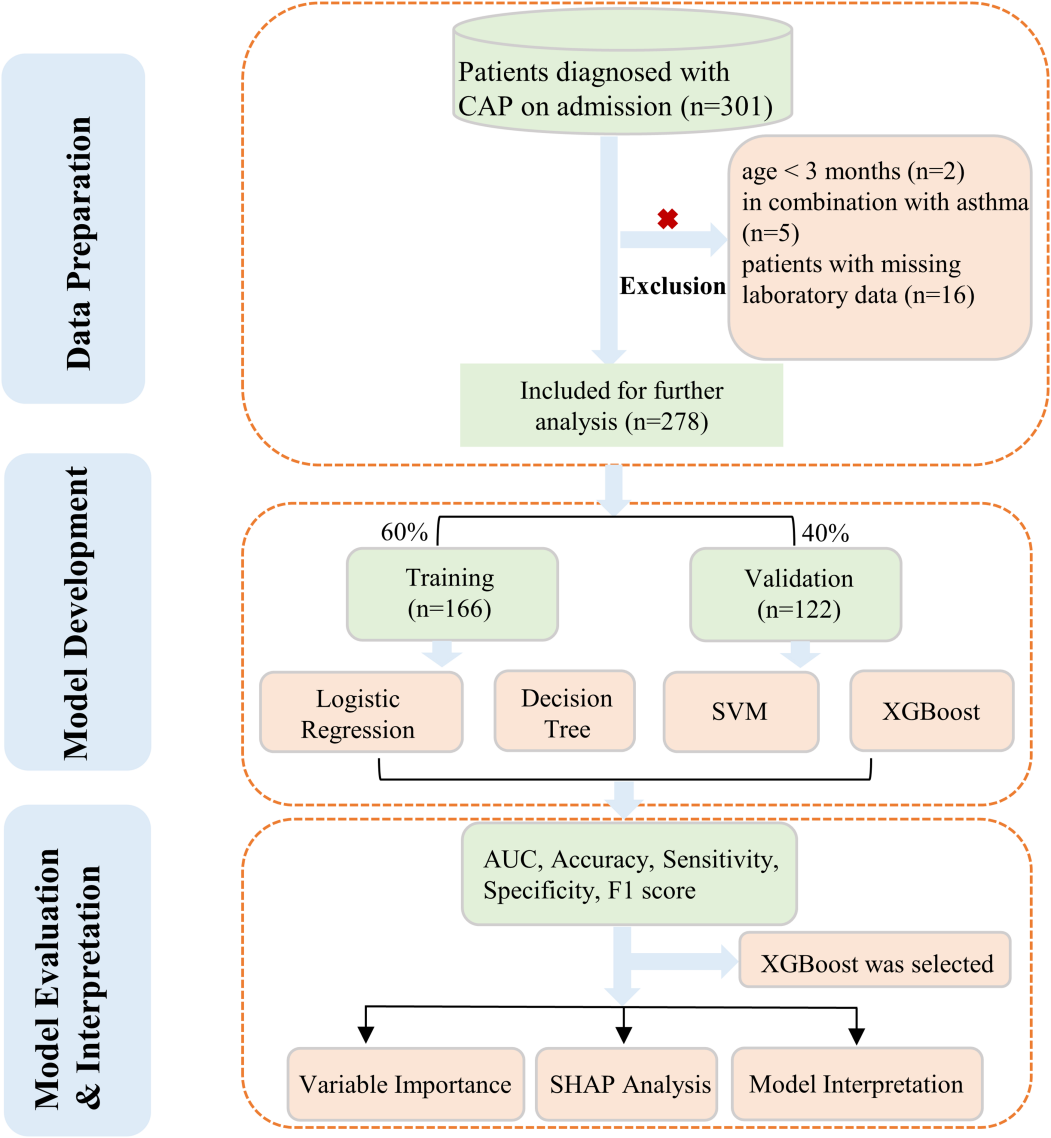
Study workflow.

Nevertheless, research focusing on the specific risk factors associated with LP in children remains limited. At present, there is a lack of information regarding the early prediction and detection of LP, and no distinct clinical laboratory or diagnostic markers for LP have been identified. This study employed four machine learning algorithms to comprehensively evaluate and quantify the risk factors associated with LP in children. The workflow was shown in Figure 1.

## 2 Methods

### 2.1 Study design and participants

A cross-sectional retrospective study was conducted on children with community-acquired pneumonia (CAP) who were admitted to the pediatric ward of Suzhou Hospital, Affiliated Hospital of Medical School, Nanjing University from January 2018 to December 2019. We included children aged under 14 years, with a diagnosis of CAP according to the guideline (5). In radiological diagnosis, pneumonia was categorized into two main types based on the pattern and extent of inflammatory infiltration visible on chest x-rays: lobar pneumonia (LP) and non-lobar pneumonia (non-LP). The diagnosis of lobar pneumonia was confirmed by an experienced attending pediatrician based on the definition provided by the guidelines (5, 12), taking into account the patient's medical history, clinical presentation, and characteristic chest imaging findings. The inclusion criteria for patients in this study were as follows: (i) patients over 3 months and under 14 years old; (ii) diagnosis of CAP; (iii) hospitalization for treatment; (iii) complete laboratory data. The study excluded patients with congenital heart disease, inherited metabolic disorders, neurological disorders, bronchopulmonary dysplasia, immunosuppression and co-infections of other systems. Within 24 h of a patient's admission, all venous blood was drawn. The study protocol was approved by the Institutional Review Board and Ethics Committee of Suzhou Hospital, Affiliated Hospital of Medical School, Nanjing University (No. IRB2021025). In consideration of the retrospective nature and anonymous analysis of the study design, the requirement for written informed consent from parents was waived.

### 2.2 Data collection and measurement

A preliminary analysis was conducted to assess the predictive characteristics associated with lobar pneumonia, considering their clinical significance and factors identified in previous studies. A total of 25 variables were included in this study. The variables consisted of patients' basic characteristics and laboratory results. Basic information included age, gender, height, weight, full term or not, delivery mode, payment method, use GCs or not, duration of fever, duration of hospitalization and prehospitalization duration. Laboratory items included white blood cells (WBC), neutrophil percentage (N%), lymphocyte percentage (L%), C-reactive protein (CRP), CD64-index, procalcitonin (PCT), prealbumin, alanine aminotransferase (ALT), aspartate aminotransferase (AST), hemoglobin (HGB), platelets (PLT), total protein (TP), albumin (ALB) and globulin (GLB).

### 2.3 Statistical analysis

Missing values were present in some variables of the dataset. These missing values in the covariates were imputed through simple imputation. Subsequent to processing, the data was randomly split into a 60% training set for model development and a 40% validation set for parameter tuning and assessing model generalizability. The detailed information between the two sets was shown in Supplementary Table S1. To evaluate the model's reliability, we utilize tenfold cross-validation on both the training and testing sets.

The univariable logistic regression and Boruta feature selection were used to perform variable filtering. First, potential risk factors screening was conducted using univariate logistic regression analysis. Factors with *P* value less than 0.05 were selected. Then, a Boruta algorithm was utilized to obtain variables from all risk features. The Boruta is a random forest-based feature selection method. It iteratively compares the importance of original variables to permuted “shadow” variables (13). Through statistical testing, it identifies and eliminates unimportant features until all variables are classified or a predefined iteration limit is reached (14). We used Boruta to assess variable importance with 1,000 iterations. The normalized hits (normHits) metric directly reflects the frequency with which a feature is deemed important across multiple iterations, serving as a straightforward indicator of feature significance (15). In general, higher normHits values correspond to greater feature importance. Additionally, the mean importance (MeanImp) value gives insight into the magnitude of a feature's importance, with elevated scores indicating higher feature relevance. Both metrics were considered to get a comprehensive view of feature importance. The final model was developed by incorporating the shared features of both algorithms.

We aimed to compare four machine learning algorithms including the Logistic Regression (LR), Support Vector Machine (SVM), Extreme Gradient Boosting (XGBoost), and Decision Tree (DT) to identify the best performing model for interpretation. To assess the machine learning algorithm's performance, a confusion matrix was utilized. Machine learning-based classifiers were assessed for area under the curve (AUC), balanced accuracy, sensitivity, specificity, and F1 score. The best-performing model, as determined by comprehensive evaluation metrics, will be selected for subsequent analysis. The significance of each feature to the model output was demonstrated using variable importance, which led to the selection of the top five variables for in-depth discussion. Moreover, a Shapley additive explanation (SHAP) was conducted for the purpose of visualizing the model.

In this study, all continuous variables were presented as the mean ± standard deviation (SD) or median and interquartile range (IQR). Categorical variables were expressed as counts and percentages. The statistical analysis process was carried out using R software (v 4.2.3), with a *P*-value of <0.05 being deemed statistically significant.

## 3 Results

### 3.1 Baseline characteristics

A total of 278 CAP pediatric patients who were hospitalized from January 2018 to December 2019 were included in the study. The dataset was relatively complete with only 2% missing values. The baseline characteristics, as presented in Table 1, were analyzed before modeling, comparing patients with non-lobar pneumonia (*n* = 213) to those with lobar pneumonia (*n* = 65). No significant differences were observed in gender distribution, full-term birth status, mode of delivery, payment method, white blood cell count, procalcitonin, prealbumin, ALT, hemoglobin, platelet count, or total protein levels between the two groups. However, lobar pneumonia group was associated with longer duration of fever (2.89 ± 2.54 vs. 3.80 ± 2.78 days, *p* = 0.014) and hospitalization (8.03 ± 1.88 vs. 6.74 ± 1.67 days, *p* < 0.001). Laboratory findings revealed that lobar pneumonia patients had significantly higher neutrophil percentage (62.80% vs. 50.80%, *p* < 0.001), C-reactive protein levels (12.00 vs. 5.00 mg/L, *p* < 0.001), CD64 index (2.45 vs. 2.13, *p* = 0.013), and globulin levels (26.60 vs. 25.00 g/L, *p* < 0.001). Conversely, they had lower lymphocyte percentage (26.90% vs. 39.00%, *p* < 0.001), AST levels (33.00 vs. 38.00 U/L, *p* = 0.009), and albumin levels (41.00 vs. 42.20 g/L, *p* = 0.003).

**Table 1:** Baseline demographic and clinical characteristics of patients with and without LP.

Characteristics	Non-lobar pneumonia (*n* = 213)	Lobar pneumonia (*n* = 65)	*P*-value
Age, years	2.62 ± 2.18	4.00 ± 2.44	<0.001
Gender			0.657
Male	105 (49.30%)	30 (46.15%)	
Female	108 (50.70%)	35 (53.85%)	
Height, cm	93.91 ± 19.73	105.68 ± 20.38	<0.001
Weight, kg	15.67 ± 6.78	19.00 ± 7.46	<0.001
Full term			0.544
No	12 (5.63%)	5 (7.69%)	
Yes	201 (94.37%)	60 (92.31%)	
Mode of delivery			0.603
Natural	145 (68.08%)	42 (64.62%)	
Cesarean	68 (31.92%)	23 (35.38%)	
Payment method			0.112
Medicare	164 (77.00%)	56 (86.15%)	
Self-payment	49 (23.00%)	9 (13.85%)	
Use GCs			0.033
No	76 (35.68%)	14 (21.54%)	
Yes	137 (64.32%)	51 (78.46%)	
Duration of fever, days	2.89 ± 2.54	3.80 ± 2.78	0.014
Duration of hospitalization, days	6.74 ± 1.67	8.03 ± 1.88	<0.001
Prehospitalization duration, days	7.54 ± 6.92	7.37 ± 6.29	0.855
White blood cell count, 10^9^/L	8.11 (6.28–11.11)	8.65 (6.98–12.02)	0.252
Neutrophil percentage, %	50.80 (35.40–63.30)	62.80 (50.60–71.40)	<0.001
Lymphocyte percentage, %	39.00 (27.80–54.90)	26.90 (18.50–42.60)	<0.001
C-reactive protein, mg/L	5.00 (3.00–11.70)	12.00 (4.00–22.00)	<0.001
CD64 index	2.13 (1.02–5.06)	2.45 (1.04–7.18)	0.013
Procalcitonin, ng/ml	0.10 (0.06–0.18)	0.10 (0.07–0.22)	0.400
Prealbumin, mg/L	147.00 (122.00–188.00)	130.00 (116.00–167.00)	0.373
ALT, U/L	13.00 (10.00–18.00)	11.00 (8.00–13.00)	0.078
AST, U/L	38.00 (32.00–48.00)	33.00 (26.00–42.00)	0.009
HGB, g/L	124.00 (118.00–130.00)	125.00 (120.00–133.00)	0.186
PLT, 10^9^/L	263.50 (212.00–321.00)	263.50 (220.00–318.00)	0.917
TP, g/L	67.60 (64.50–70.60)	67.70 (64.70–70.90)	0.346
ALB, g/L	42.20 (40.50–44.10)	41.00 (38.90–43.80)	0.003
GLB, g/L	25.00 (22.90–27.60)	26.60 (24.50–30.20)	<0.001

### 3.2 Feature selection

**Figure 2:**
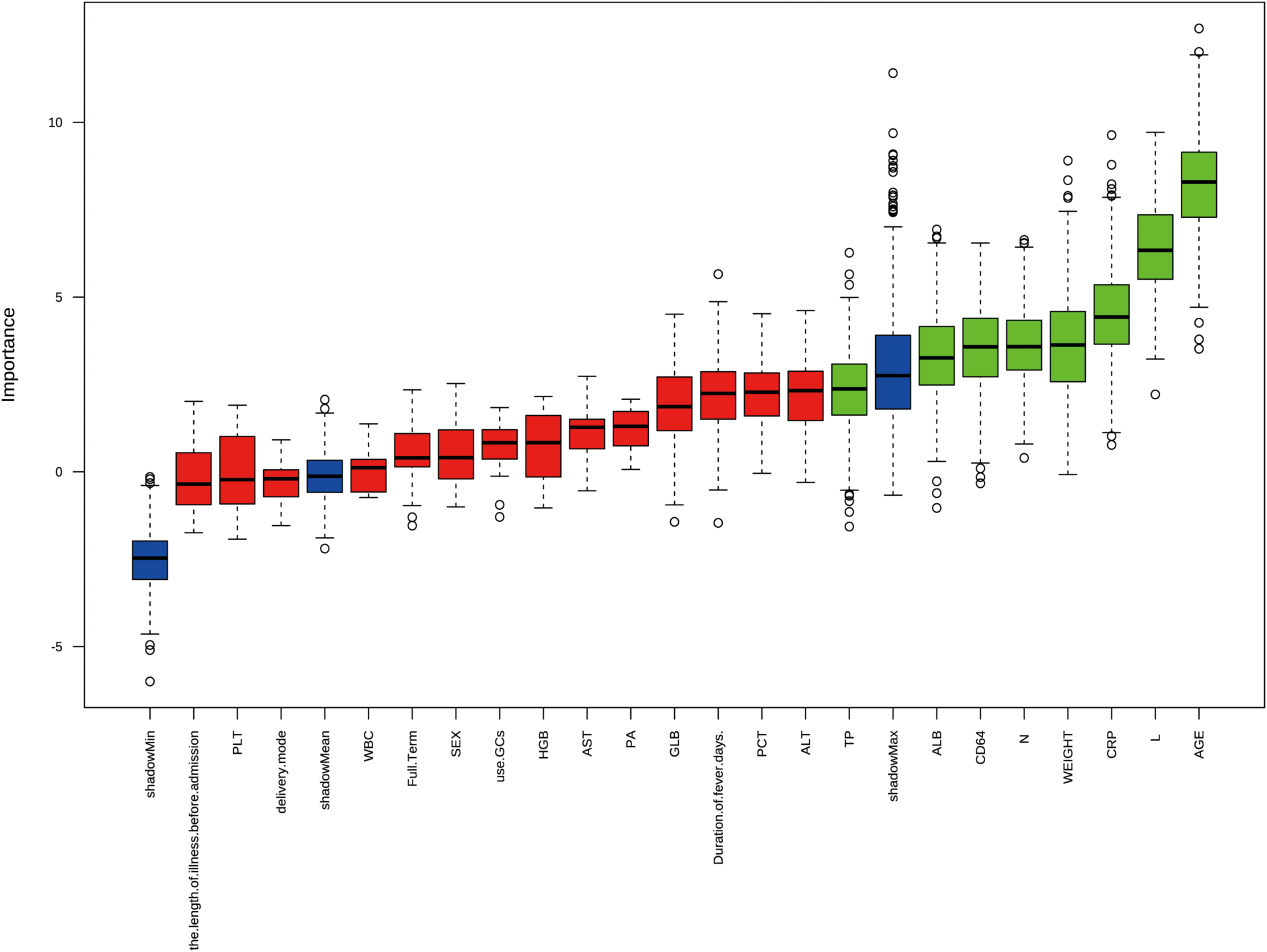
Feature selection results using boruta algorithm from 22 initial clinical variables. The boxplot shows the *Z*-score importance of each variable after 1,000 iterations, where green boxes represent confirmed important features, and red boxes represent rejected features. The *y*-axis represents the importance score, and variables are ordered by their relative importance on the *x*-axis.

The univariable logistic regression analysis (Table 2) was conducted to select several significant predictors that associated with lobar pneumonia (LP). Age (OR = 1.356, 95%CI: 1.156–1.606), weight (OR = 1.080, 95%CI: 1.025–1.141), and duration of fever (OR = 1.203, 95%CI: 1.050–1.387) were positively associated with LP. Inflammatory markers, CRP (OR = 1.021, 95%CI 1.006–1.039) and neutrophil percentage (OR = 1.035, 95%CI: 1.012–1.061) showed positive associations, while lymphocyte percentage demonstrated a negative correlation (OR = 0.952, 95%CI: 0.925–0.977) with LP. Regarding biochemical parameters, ALB, ALT, and AST are negatively associated with LP. Additionally, CD64 index (OR = 1.059, 95%CI: 1.003–1.124) and GLB (OR = 1.115, 95%CI: 1.013–1.231) showed significant positive associations. All reported features were statistically significant at the *p* < 0.05 level. Figure 2 showed the results of feature selection based on the Boruta algorithm. After 1,000 iterations, the variables were sorted based on their *Z* score value. The green variables, including age, lymphocyte percentage, CRP, weight, neutrophil percentage, CD64 index, ALB, and TP were deemed acceptable. The variable known as “age” had a meanImp score of 12.69 and a maximum normHits value that can reach 0.98. Considering the above two approaches and incorporating clinical expertise from experienced physicians, the variables age, weight, duration of fever, CRP, lymphocyte percentage, neutrophil percentage, CD64 index, ALT, AST, and ALB were selected for building the machine learning models.

**Table 2:** Univariable logistic regression for identifying risk factors associated with LP.

Characteristics	*β*	SE	OR, 95%CI	*P*-value
Age, years	0.304	0.08325	1.356 (1.156–1.606)	0
Weight, kg	0.077	0.0272	1.08 (1.025–1.141)	0.005
Duration of fever, days	0.185	0.07031	1.203 (1.05–1.387)	0.009
CRP, mg/L	0.021	0.00822	1.021 (1.006–1.039)	0.012
Lymphocyte percentage	−0.049	0.01375	0.952 (0.925–0.977)	0
Neutrophil percentage	0.035	0.01182	1.035 (1.012–1.061)	0.004
ALB, g/L	−0.167	0.06251	0.847 (0.746–0.955)	0.008
CD64 index	0.058	0.0282	1.059 (1.003–1.124)	0.041
ALT, U/L	−0.078	0.03615	0.925 (0.855–0.985)	0.032
AST, U/L	−0.035	0.01682	0.966 (0.933–0.997)	0.038
GLB, U/L	0.108	0.04943	1.115 (1.013–1.231)	0.028

### 3.3 Model performance comparisons

**Figure 3:**
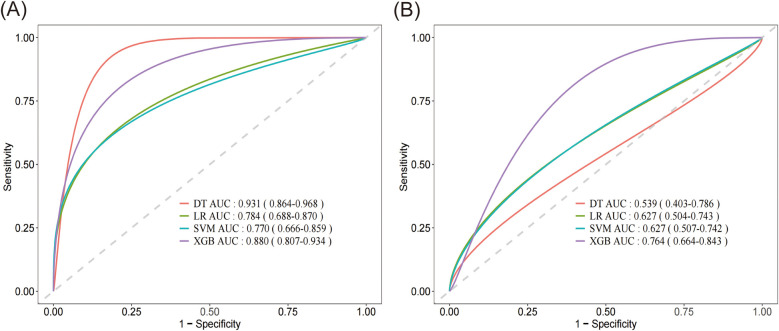
Smoothed receiver operating characteristic (ROC) curve graph of four different machine learning models. **(A)** Comparison of the AUC performance metrics among DT, LR, SVM and XGBoost algorithms on the training set. **(B)** Comparison of the AUC performance metrics among DT, LR, SVM and XGBoost algorithms on the validation set. DT, decision tree; LR, logistic regression; SVM, support vector machine; XGB, XGBoost.

Based on Figure 3, the smoothed Receiver Operating Characteristic (ROC) curve graph compares the performance of four different machine learning models. The graph displays ROC curves for Logistic Regression (LR), Support Vector Machine (SVM), XGBoost model, and Decision Tree (DT). The *x*-axis represents 1-specificity, and the *y*-axis represents sensitivity, with curves closer to the upper left corner indicating better model performance. In the training set, the DT model exhibited the highest predictive capability with an AUC of 0.931, with a 95% confidence interval (CI) of 0.864–0.968, closely followed by the XGBoost model with an AUC of 0.880 (95% CI: 0.807–0.934). The LR and SVM model demonstrated relatively lower performance in the training set, achieving AUC values of 0.784 (95% CI: 0.668–0.870) and 0.770 (95% CI: 0.666–0.859), respectively. In contrast, within the validation set, the XGBoost model maintained superior predictive capability with an AUC of 0.764 (95% CI: 0.664–0.843), significantly outperforming the other algorithms. Both LR and SVM achieved identical AUC values of 0.627, accompanied by similar 95%CI (LR: 0.504–0.743; SVM: 0.507–0.742). The DT model exhibited the lowest performance in the validation set, with an AUC of 0.539 (95% CI: 0.403–0.786).

We evaluated the performance of four ML algorithms using AUC value and confusion matrix-derived metrics (accuracy, sensitivity, specificity, and F1 score) across both training and validation datasets (Table 3). Among all algorithms, the XGBoost model demonstrated the most consistent and superior performance, with robust training metrics (AUC 0.880, accuracy 0.819, specificity 0.916) and maintained relatively stable performance in validation (AUC 0.764, accuracy 0.611, specificity 0.878). While the DT model initially showed the highest training performance (AUC 0.931, accuracy 0.811, specificity 0.930), it experienced significant performance degradation in validation (AUC 0.539, accuracy 0.633, specificity 0.789), suggesting potential overfitting. LR and SVM demonstrated similar performance metrics, both of which were inferior to XGBoost. Overall, the XGBoost model displayed the most consistent and robust performance across both datasets, making it the superior choice for predictive modeling in our study.

**Table 3:** Evaluation of the performance of four algorithms based on AUC and confusion matrix.

Algorithm	Data set	AUC	Balanced accuracy	Sensitivity	Specificity	F1 score
LR	Train	0.784	0.759	0.778	0.740	0.571
	Valid	0.627	0.660	0.552	0.768	0.500
SVM	Train	0.770	0.763	0.694	0.832	0.602
	Valid	0.627	0.598	0.379	0.817	0.400
XGBoost	Train	0.880	0.819	0.722	0.916	0.712
	Valid	0.764	0.611	0.345	0.878	0.408
DT	Train	0.931	0.811	0.692	0.930	0.720
	Valid	0.539	0.633	0.476	0.789	0.400

### 3.4 Model interpretation

**Figure 4:**
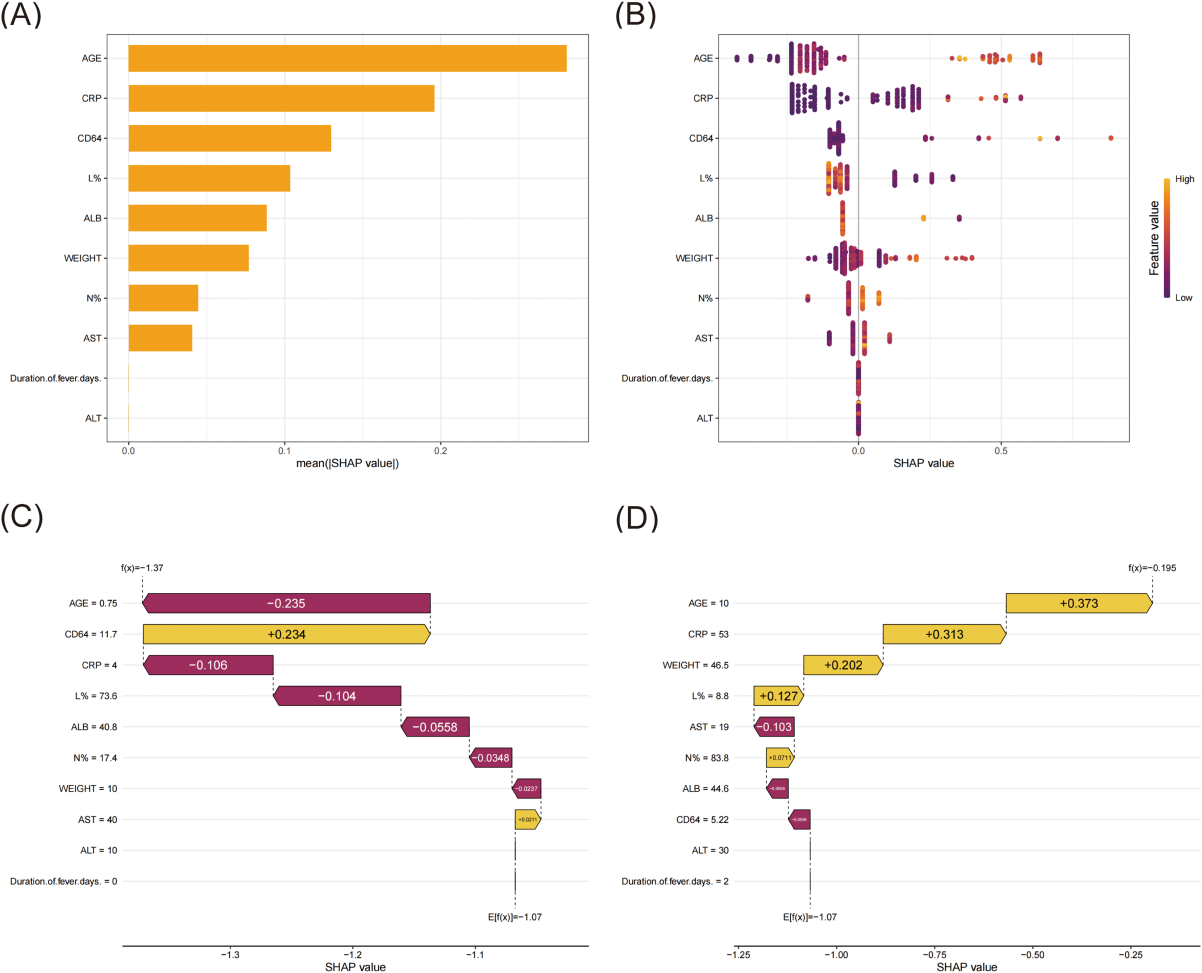
SHAP interpret the model. **(A)** Ranking of variable importance based on the mean SHAP value. **(B)** Beeswarm plots of feature distribution. All training cases were used to illustrate the features, with each row representing a feature and the *X*-axis showing the SHAP value. The distribution of SHAP values for each feature was displayed through color coding, where yellow indicated higher feature values and magenta indicated lower feature values. **(C)** SHAP waterfall for case one. **(D)** SHAP waterfall for case two.

As the XGBoost algorithm proved to have the best predictive performance, we employed SHAP analysis to interpret the model and identify risk factors associated with pediatric lobar pneumonia (Figures 4A,B). According to the SHAP value, age, CRP, CD64 index, L%, and ALB emerged as the top five predictive factors. Case one (Figure 4C) involved a 9-month-old (0.75 years) infant weighing 10 kg. SHAP waterfall analysis revealed that the model's final output value f(x) = −1.37 was lower than the baseline prediction E[f(x)] = −1.07, indicating a reduced likelihood of lobar pneumonia. Among the contributing factors, age (0.75 years) demonstrated the strongest negative contribution (SHAP value: −0.235), followed by CRP (4 mg/L, −0.106) and lymphocyte percentage (73.6%, −0.104). Additional negative contributions were observed from ALB (40.8 g/L, −0.0558), neutrophil percentage (17.4%, −0.0348), and weight (10 kg, −0.0237). In contrast, two variables showed positive contributions: CD64 index (11.7, + 0.234) and AST (40 U/L, +0.0211). Case two (Figure 4C) involved a 10-year-old patient weighing 46.5 kg. SHAP waterfall analysis revealed that the model's final output value f(x) = −0.195 was higher than the baseline prediction E[f(x)] = −1.07, suggesting an increased likelihood of lobar pneumonia. Among the contributing factors, age (10 years) exhibited the strongest positive contribution (SHAP value: +0.373), followed by CRP (53 mg/L, +0.313) and weight (46.5 kg, +0.202). Additional positive contributions were lymphocyte percentage (8.8%, +0.127) and neutrophil percentage (83.8%, +0.0711). In contrast, three features showed negative contributions: AST (19 U/L, −0.103), ALB (44.6 g/L, −0.0558), and CD64 index (5.22, −0.0556).

## 4 Discussion

Over the past few years, the prevalence of lobar pneumonia among children has been on the rise, presenting a substantial challenge for its early clinical identification and treatment (16). The clinical presentation of lobar pneumonia in children is frequently atypical at the outset, primarily characterized by symptoms such as fever, cough, and other respiratory manifestations. Initial lung auscultation can be challenging in detecting the lesions, often leading to confusion with upper respiratory tract infections, acute tracheitis, and other similar conditions. If left undetected and untreated in its early stages, the condition can rapidly progress to complications including empyema, pyopneumothorax, pleural effusion, lung atelectasis, lung necrosis, and extrapulmonary complications such as liver dysfunction, myocardial damage, and gastrointestinal dysfunction. In some cases, it may evolve into severe, refractory pneumonia, or even multiple organ failure, posing a significant threat to life. Consequently, early identification, precise diagnosis, and prompt treatment are of utmost importance.

In this retrospective cohort study, we developed and evaluated four machine learning models to predict the risk of LP in pediatric patients with CAP. Among these models, XGBoost demonstrated the most stable performance, outperforming logistic regression, support vector machine, and decision tree models, with an AUC of 0.880, accuracy of 0.819, and specificity of 0.916 in the training set, and an AUC of 0.764, accuracy of 0.611, and specificity of 0.878 in the validation set. SHAP analysis identified age, CRP, CD64 index, lymphocyte percentage, and albumin as the five most important predictive factors. The local interpretability of the model was further confirmed through case studies, where older age, elevated CRP, and altered lymphocyte percentages were found to have a significant impact on predicting the risk of LP. These findings suggest that the XGBoost model holds potential value for the early identification of LP in pediatric CAP patients, aiding in timely clinical decision-making.

This study revealed that the age in the lobar pneumonia group was 4.00 ± 2.44 years, markedly older than that observed in the non-lobar pneumonia group (2.62 ± 2.18 years), aligning with prior research conducted by other scholars (16). Older children possess more robust natural immune systems and relatively mature self-protective mechanisms. Their own antibodies can restrict lung inflammation to specific lobes or segments, whereas younger children, with less developed immune systems, often experience more widespread lung inflammation affecting multiple lobes or segments. Recent studies have found that the CD64 index, a diagnostic indicator for infectious diseases, has been widely used in the diagnosis of sepsis, systemic infection, bronchitis, and acute pancreatitis (17, 18). A previous study found that the CD64 index exhibited superior diagnostic value compared to conventional markers (WBC, PCT and CRP) in early detection of neonatal infections, suggesting its potential as a reliable biomarker for early diagnosis (19). However, Gros A. et al. found that although the CD64 index demonstrates high specificity (89%) in diagnosing bacterial infections in ICU patients, its relatively low sensitivity indicates that the marker may be more suitable for combined application with other biological markers rather than standalone use. CRP, an acute-phase reactant produced by the liver, plays a pivotal role in inflammatory processes and exhibits high sensitivity in the clinical diagnosis of infectious diseases. It is frequently utilized in clinical settings as a non-specific marker to evaluate infection, the intensity of inflammatory responses, tissue damage, and patient prognosis (20, 21). Research has indicated that hypoalbuminemia constitutes an independent risk factor for an unfavorable prognosis in patients suffering from severe community-acquired pneumonia (22). This condition may be associated with factors such as inflammatory responses, inadequate nutritional status, and diminished immune function, all of which can influence the recovery process of pneumonia.

Furthermore, hypoalbuminemia might also correlate with the severity of lung infection and the magnitude of inflammatory responses (23). Our findings align with this, showing that children with lobar pneumonia exhibit lower albumin levels compared to those with non-lobar pneumonia, while concurrently presenting higher CRP levels in the former group. Neutrophils and lymphocytes are pivotal in the immune response to lobar pneumonia. Neutrophils, a category of white blood cells, are the initial line of defense against inflammation, often surging in response to infection or tissue injury. The activation and accumulation of neutrophils in the lungs are associated with adverse clinical outcomes, as it indicates a suppressed immune state that may impair the patient's capacity to eradicate infections. Research has established that a diminished lymphocyte ratio heightens the mortality risk in pneumonia patients (24), a trend mirrored in our study, where children with lobar pneumonia exhibit a lower lymphocyte ratio than those with non-lobar pneumonia. This disparity could be attributed to the direct assault on lymphocytes by pathogens, heightened lymphocyte consumption due to immune system activation, or the migration of lymphocytes from the bloodstream to the infection site.

A recent single-center retrospective study enrolled 533 pediatric patients with LP caused by *Mycoplasma pneumoniae (MP)* infection, aiming to analyze clinical characteristics and establish a predictive model for bronchoscopic intervention (25). Through binary logistic regression analysis, the authors identified risk factors for bronchoscopic intervention, including fever duration ≥6.5 days before bronchoscopy, CRP ≥20.94 mg/L, LDH ≥461.5 U/L, fever, and pleural effusion. The predictive scoring model based on these factors indicated that patients with scores ≥6 points had >80% probability of requiring bronchoscopic intervention. The model demonstrated an area under the ROC curve of 0.860 (95% CI: 0.824–0.897) (25). Compared with the previous research, our study demonstrated both unique contributions and complementary findings. While Li. et al*. (*25) developed a scoring system for predicting bronchoscopic intervention in *MP*-induced lobar pneumonia with comparable model performance (AUC 0.860), our machine learning approach, particularly the XGBoost model (AUC 0.880 for training set and 0.764 for validation set). Additionally, both studies identified CRP as a crucial predictor. However, our study incorporated novel biomarkers such as CD64 index and emphasized the role of age, lymphocyte percentage, and ALB. Furthermore, our machine learning approach, validated through both training and validation sets, provides a more robust framework for risk prediction compared to the conventional logistic regression analysis (26). While their scoring system offers practical clinical utility with specific cutoff points, our model's ability to predict LP risk in CAP patients enables earlier intervention.

In this study, we compared four machine learning algorithms (LR, SVM, XGBoost, and DT) for predicting pediatric lobar pneumonia. The XGBoost model demonstrated superior and more stable performance compared to other algorithms, achieving an AUC of 0.880 in the training set and maintaining robust performance with an AUC of 0.764 in the validation set. While the DT model showed the highest AUC (0.931) in the training set, its significant performance drop in the validation set (AUC 0.539) suggested overfitting. In addition, to enhance model interpretability, we employed SHAP analysis to identify and rank the contributing factors. The analysis revealed that age, CRP, CD64 index, lymphocyte percentage, and ALB were the top five influential risk factors. We also generated SHAP dependence plots for each variable, which are provided in the Supplementary Figure S1. Through case-specific SHAP waterfall analysis, we observed that these features contributed differently to individual predictions. For instance, older age, elevated CRP levels, and higher neutrophil percentage typically indicated an increased likelihood of LP, while factor such as higher ALB levels were associated with reduced risk. Comparatively, other studies have also explored the application of machine learning in pediatric bacterial infections, yielding promising results. For instance, Kanwal et al*.* (27) utilized photoplethysmography (PPG) signals and machine learning classifiers to diagnose CAP in children, achieving high accuracy in low-resource settings, which highlights the potential of non-invasive diagnostic tools. Similarly, Chiu et al. (28) demonstrated that machine learning models, including XGBoost, outperformed traditional scoring systems in predicting invasive bacterial infections (IBIs) in febrile infants, with AUROC values reaching 0.85. Le et al. (29) applied machine learning to predict severe sepsis in pediatric patients using electronic health record data, achieving an AUROC of 0.916 at the time of onset, significantly outperforming conventional scoring systems. Furthermore, Liu et al*.* (30) developed machine learning models to predict ICU admission for pediatric pneumonia patients, emphasizing the importance of timely decision-making in critical care. A recent study by a Taiwanese group (31) introduced an explainable deep learning model for predicting IBIs in febrile infants, achieving an AUROC of 0.87 while providing interpretability through SHAP analysis. These studies collectively underscore the growing role of machine learning in the management of pediatric bacterial infections. Our findings align with this trend, contributing to the advancement of predictive tools for pediatric bacterial infections while specifically addressing the unique challenges of predicting lobar pneumonia.

This study has several limitations. First, the relatively small sample size (278 patients with CAP, including 65 LP cases) may limit the model's robustness and generalizability. This sample size is notably smaller compared to some other machine learning studies in pediatric infections, such as Chiu et al*.*' s study (28) with 4,211 patients and Liu et al.'s study (30) with 8,464 cases. The limited sample size might affect the model's performance. In addition, the relatively homogeneous study population from a single center may not fully represent pediatric populations from different regions or with varying environmental factors. Although chest x-ray was essential for the initial categorization of pneumonia types in our study, our model excluded radiological features in favor of immediately available clinical and laboratory parameters at admission, which may have limited the model's comprehensive predictive capability. While our model demonstrated good performance in the training set (AUC 0.880), the moderate decrease in performance in the validation set (AUC 0.764) suggests potential variability in model generalization. The top five risk factors identified through SHAP analysis (age, CRP, CD64 index, lymphocyte percentage, and ALB) may vary in their predictive strength across different populations and clinical settings. Additionally, certain laboratory parameters used in our model, particularly the CD64 index, may not be routinely available in all healthcare institutions, which could limit the model's widespread application. Furthermore, differences in clinical practices, diagnostic criteria, and treatment protocols across institutions may impact the model's performance and applicability. Future studies should focus on external validation through multi-center and multi-regional cohorts to assess the model's robustness across different clinical settings and populations.

## 5 Conclusion

In conclusion, our study developed and compared four machine learning models for predicting lobar pneumonia in children with CAP. Among the models, the XGBoost algorithm demonstrated the best overall performance. The model achieved an AUC of 0.880 in the training set and 0.764 in the validation set, generally outperforming LR, SVM, and DT models.

SHAP analysis was employed to interpret the XGBoost model, identifying age, CRP, CD64 index, lymphocyte percentage, and albumin (ALB) as the top five predictive factors for LP. These findings not only validate the importance of traditional clinical indicators but also highlight the potential of machine learning to uncover additional predictive insights. Despite the limitations of this single-center, small-sample retrospective study, our findings suggest that the XGBoost model provides a reliable tool for early diagnosis and risk assessment of LP in children.

## Data Availability

The raw data supporting the conclusions of this article will be made available by the authors, without undue reservation.
