# Supplementary figures and images for: Advancing risk factor identification for pediatric lobar pneumonia: the promise of machine learning technologies
Source: Front Pediatr. 2025 Mar 7;13:1490500. doi: 10.3389/fped.2025.1490500 (PMC11925904; doi:10.3389/fped.2025.1490500)

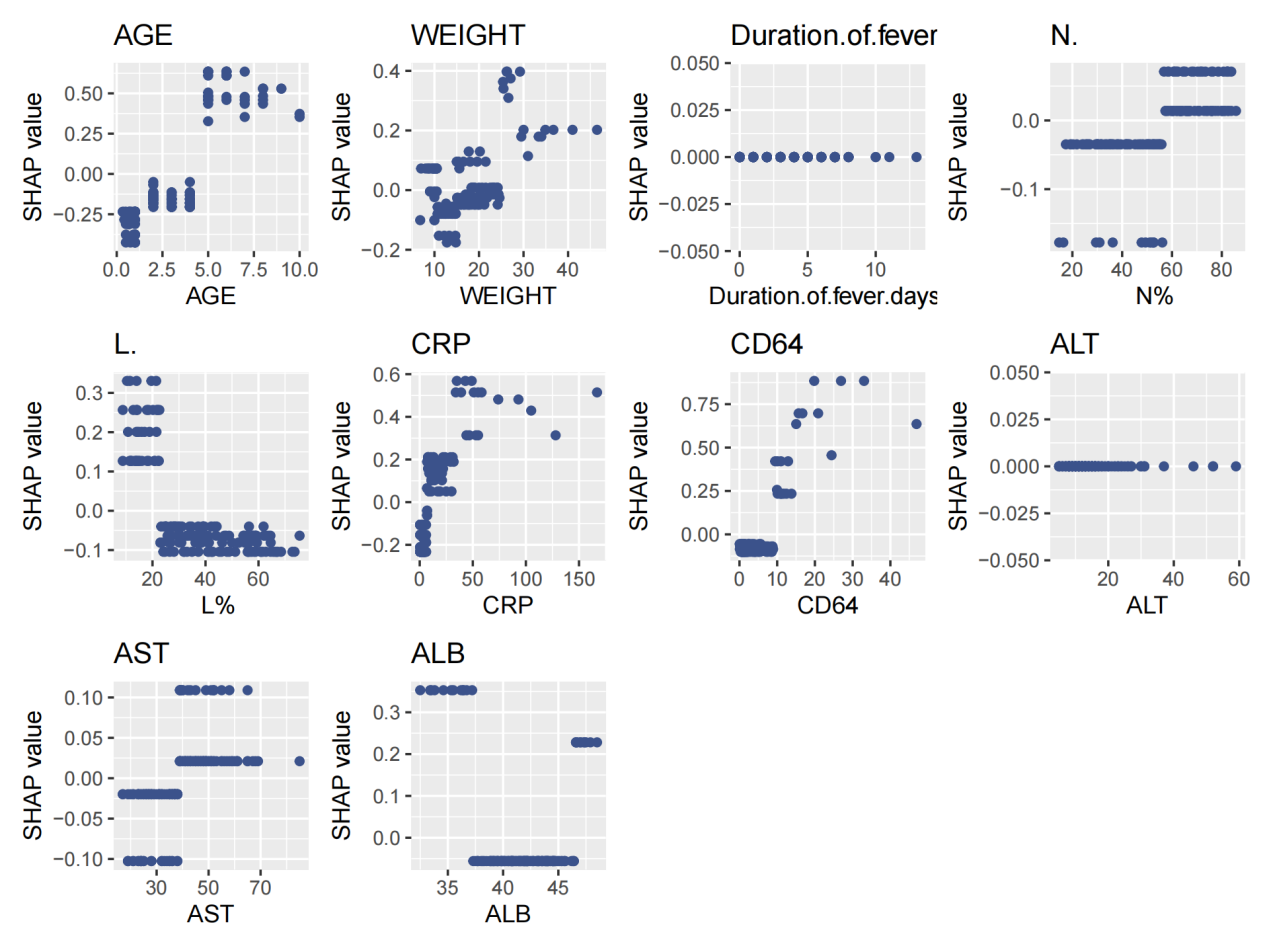


**Figure S1 SHAP feature dependence plots based on XGBoost Model.**

Supplement: Supplementary file 2 [file Datasheet1.docx]
